# Letter to the Editor 

**Published:** 2018

**Authors:** Tadaki Nakahara

**Affiliations:** MD, PhD, Department of Radiology, Keio University School of Medicine, Tokyo, Japan

We read with interest the article by Tsutsui et al. (Tsutsui Y, Awamoto S, Himuro K, Umezu Y, Baba S, Sasaki M.: Characteristics of Smoothing Filters to Achieve the Guideline Recommended Positron Emission Tomography Image without Harmonization. Asia Ocean J Nucl Med Biol. 2018 Winter; 6(1):15-23. doi: 10.22038/aojnmb.2017.26684.1186.) (1). However, we were really stunned by the absence of the citation of our recent work (2) regarding a technical approach for nuclear image harmonization.

Although our study dealt with SPECT but not PET, we found two major common harmonization methodologies between our and their studies: (a) comparison of actual NEMA phantom image and digital reference object (DRO) developed by the Quantitative Imaging Biomarker Alliance (QIBA) FDG PET technical committee, and (b) use of the root mean squared error (RMSE) for evaluation of appropriate harmonization. The formula and figure of RMSE in their article were quite similar to ours.

Of key importance, the use of the DRO was firstly published in 2015 (3), only analyzing calculation accuracy of PET standardized uptake value. Since then, to our knowledge, our study would be the first to show a harmonization procedure of nuclear image quantitation using the DRO. 

Given this, we believe that the authors should have cited our work. 

## Conflicts of interest

None declared.
